# Supplementary material for: Identification of photoperiod-induced specific miRNAs in the adrenal glands of Sunite sheep (Ovis aries)
Source: Front Vet Sci. 2022 Jul 22;9:888207. doi: 10.3389/fvets.2022.888207 (PMC9354845; doi:10.3389/fvets.2022.888207)
Supplement: Supplementary file 1 [file Data_Sheet_1.ZIP › Supplementary file/miRDeep2 file.zip!/miRDeep2 file/Know_Structure/LP42A3/oar-let-7f.pdf]

miRBase precursor : oar-let-7f
Total read count : 577884
oar-let-7f read count : 577739
remaining reads : 145

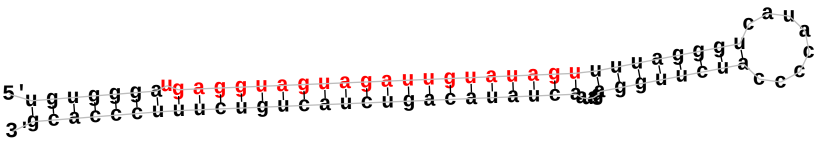

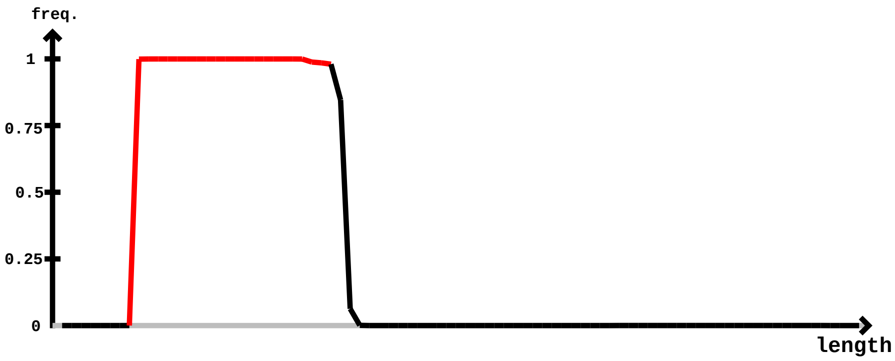


```
        oar-let-7f
5'- ugugggaugagguaguagauuguauaguuuuagggucauaccccaucuuggagauaacuauacagucuacugucuuucccacg   -3'    exp
    .((((((.(((((((((((((((((((((((((((((.......))))))))....))))))))))))))))))))))))))).   reads      mm         sample
    ......augagguaguagauuguaua.........................................................   1          0          seq
    ......augagguaguagauuguauag........................................................   1          0          seq
    ......augagguaguagauuguauagu.......................................................   5          0          seq
    ......Gugagguaguagauuguauagu.......................................................   1          1          seq
    ......augagguaguagauuguauaguu......................................................   28         0          seq
    ......Gugagguaguagauuguauaguu......................................................   3          1          seq
    ......augagguaguagauuguauaguuu.....................................................   3          0          seq
    .......ugagguaguagauuguU...........................................................   7          1          seq
    .......ugagguaguagauugAa...........................................................   1          1          seq
    .......ugagguGguagauugua...........................................................   1          1          seq
    .......ugaAguaguagauugua...........................................................   3          1          seq
    .......ugagguaguagauugua...........................................................   183        0          seq
    .......ugagguaguUgauuguau..........................................................   6          1          seq
    .......ugagguaguagauuguUu..........................................................   5          1          seq
    .......ugagguaguagauuguau..........................................................   6503       0          seq
    .......ugaAguaguagauuguau..........................................................   44         1          seq
    .......ugagguaguagauuguaua.........................................................   1724       0          seq
    .......ugagguaguagauuguauag........................................................   2880       0          seq
    .......ugagguaguagauuguauagu.......................................................   77485      0          seq
    .......ugagguaguagauugAauagu.......................................................   13         1          seq
    .......ugagguaguagauuguauaguu......................................................   452208     0          seq
    .......ugagguaguagauuguauaguuu.....................................................   34319      0          seq
    .......ugagguaguagauuguauaguuG.....................................................   1527       1          seq
    .......ugagguaguagauuguauaguuGu....................................................   96         1          seq
    .......ugagguaguagauuguauaguuuu....................................................   318        0          seq
    .......ugagguaguagauuguauaguuuua...................................................   5          0          seq
    ........gagguaguagauuguau..........................................................   6          0          seq
    ........gagguaguagauuguaua.........................................................   1          0          seq
    ........gagguaguagauuguauagu.......................................................   47         0          seq
    ........gagguaguagauuguauaguu......................................................   238        0          seq
    ........gagguaguagauuguauaguuu.....................................................   13         0          seq
    ........gagguaguagauuguauaguuG.....................................................   2          1          seq
    .........agguaguagauuguauagu.......................................................   2          0          seq
    .........agguaguagauuguauaguu......................................................   7          0          seq
    .........agguaguagauuguauaguuG.....................................................   2          1          seq
```

oar-let-7f

```
ugugggaugagguaguagauuguauaguuuuagggucauaccccaucuuggagauaacuauacagucuacugucuuucccacg
.........agguaguagauuguauaguuu.....................................................    3      0     seq
.........agguaguagauuguauaguuuu....................................................    1      0     seq
.........agguaguagauuguauaguuGu....................................................    43     1     seq
..........gguaguagauuguauaguu......................................................    1      0     seq
..........gguaguagauuguauaguuG.....................................................    1      1     seq
...........guaguagauuguauaguuuu....................................................    1      0     seq
.............aguagauuguauaguuG.....................................................    1      1     seq
.............................uuagggucauaccccaucuugga...............................    2      0     seq
.............................uuagggucauaccccaucuuggaga.............................    2      0     seq
..........................................ccaucuuggagauaacuauac....................    1      0     seq
.....................................................auaacuauacagucuacugucu........    1      0     seq
......................................................uaacuauacagucuacugucuuu......    1      0     seq
.......................................................aacuauacagucuacugucuuu......    1      0     seq
.......................................................aacuauacagucuacugucuuuc.....    1      0     seq
.........................................................cuauacagucuacugucuuu......    6      0     seq
.........................................................cuauacagucuacugucuuuc.....    23     0     seq
.........................................................cuauacagucuacugucuuucc....    107    0     seq
```
